# Sex differences in coronary artery bypass grafting-related morbidity and mortality

**DOI:** 10.3389/fcvm.2022.1021363

**Published:** 2022-11-29

**Authors:** Jouko Nurkkala, Anni Kauko, Joonatan Palmu, Jenni Aittokallio, Teemu Niiranen

**Affiliations:** ^1^Department of Anesthesiology and Intensive Care, University of Turku, Turku, Finland; ^2^Division of Perioperative Services, Intensive Care and Pain Medicine, Turku University Hospital, Turku, Finland; ^3^Department of Internal Medicine, University of Turku, Turku, Finland; ^4^Division of Medicine, Turku University Hospital, Turku, Finland; ^5^Department of Public Health Solutions, Finnish Institute for Health and Welfare, Helsinki, Finland

**Keywords:** sex-difference, women, survival, morbidity, coronary artery bypass grafting

## Abstract

**Background:**

Coronary artery bypass grafting (CABG) is associated with both cardiovascular disease (CVD) and non-CVD traits. In addition, women’s prognosis after coronary events and revascularizations is worse than in men. As the course of CVD in women differs from that of men, we performed a phenome-wide analysis on the sex differences in CABG -related morbidity and mortality.

**Materials and methods:**

We performed an untargeted analysis on the sex differences in predictors and outcomes of CABG. We studied a sample of 176,680 FinnGen participants, including 5,950 individuals who underwent CABG (4,988 men and 962 women) and were followed between 1998 and 2019. Over 1,100 different traits were analyzed for both sexes and the results were adjusted with age, smoking status and BMI. Cox proportional hazards models with sex-trait interactions were used to estimate the associations between (1) traits and incident CABG; and (2) CABG and incident traits.

**Results:**

In women, CABG was more strongly related to greater increases in risk of diseases such as hypertension, Alzheimer’s, aortic aneurysms, gout, and chronic kidney disease compared to risk increases observed in men (all interaction *p*-values < 0.03). After CABG, men had 2.5-fold (*p* = 3.1E−15) and women 6.3-fold (*p* = 9.4E−08) greater risk of cardiac death compared to same-sex individuals who did not undergo CABG (p for interaction 8.2E−4). Moreover, the risk of death in women remained higher even 12 years after CABG, whereas the long-term risk of death in men was not increased, compared to same-sex individuals who did not undergo CABG.

**Conclusion:**

The adverse outcomes after CABG, both quantity and quality, also appear to differ between men and women. In women, CABG is related to greater long-term increases in risk of cardiac death and several other disease states than in men. Consideration should therefore be given to whether women receive adequate long-term post-operative therapy and follow-up as CABG is not associated with equally improved cardiovascular disease prognosis in women than in men.

## Introduction

Outcomes of coronary heart disease (CHD) and coronary artery bypass crafting (CABG) have constantly improved over the past decades (1–3). However, women’s prognosis after coronary events and revascularizations, including CABG, still remain markedly impaired compared to that of men (4, 5). The mechanisms explaining this observed sex difference are undoubtedly multifactorial and partially related to older age at the time of surgery and greater comorbidity (6). Especially diabetes and hypertension are more common in women with CHD and are related to increased risk in women compared to men (6–10). Also, women with CHD and acute coronary syndrome often present themselves with atypical symptoms which may lead to a delayed diagnosis and treatment, resulting in worse outcomes (11, 12).

The obvious benefits of CABG are significantly improved quality of life (13) and decreased mortality (4, 14, 15), but conversely, the procedure exposes the patient to several other late post-operative comorbidities and even unexpected conditions such as depression (16). Most previous studies have assessed the impact of CABG on cardiovascular disease (CVD) morbidity (17–19), but CABG is also found to associate with several non-CVD traits such as anemia, gastrointestinal traits, septicemia, lung cancer, Alzheimer’s disease and chronic obstructive pulmonary disease (20), which may be reflective of the shared comorbidities between these diseases and CHD. Despite the known sex-differences in the course of CHD, the definite reason for the poorer survival after coronary procedures in women remains unclear, highlighting the urgent need for further studies.

Some sex differences in the CABG-related morbidity are known to exist (5). To our knowledge, however, no phenome-wide untargeted analysis of the sex differences in traits that are associated with future and prior CABG, including non-CVD traits, has been performed. Thus, we investigated the sex-dependent differences in CABG correlates, which could further explain the observed sex differences in CABG morbidity and mortality. To address the question, we considered health data of >300,000 participants of the FinnGen study and then performed a systematic analysis of >1,100 CABG predictors and outcomes in men and women.

## Materials and methods

### Study sample

The original study sample comprised 309,154 individuals from the FinnGen Data Freeze 7 which included Finnish participants from national hospital biobanks, prospective epidemiologic studies, and disease-based cohorts. All participants in the FinnGen study were linked to data from the nationwide National Hospital Discharge, Cause of Death, Cancer, and Medication Reimbursement registers by using personal identification codes.

From the original study sample 132,474 individuals with missing BMI and smoking data were excluded from the study and thus the final study sample consisted of 176,680 individuals (82,794 men and 91,568 women). Of these participants, 5,950 (4,988 men and 962 women) individuals underwent CABG surgery during follow-up. All study participants provided an informed written consent before their participation in the FinnGen study. This study protocol was approved by The Coordinating Ethical Committee of the Hospital District of Helsinki and Uusimaa, as described in the Supplementary material.

### Register-based traits and follow-up

The analysis period extended from January 1, 1998, to December 31, 2019. The predictor- and outcome-traits were defined by ICD and ATC codes as described in the Supplementary Table 1. In total, 4,182 different traits with incidence *n* ≥ 1 were defined in the FinnGen study and thus formed 8,364 pairs of events (traits preceding CABG and CABG preceding traits). However, event pairs with any subgroup size of less than 10 individuals were excluded from the study to comply with the privacy protocol of the FinnGen study.

### Statistical analysis

We used a case-cohort design with Cox’s regression models to study the associations between different traits and CABG in men and in women separately (21). The cohort size was 10,000. Cox’s regression models with sex-trait interaction term were performed separately for (1) each predictor trait and CABG and (2) for each CABG and outcome trait. We adjusted *p*-values for multiple testing and false discovery rate (FDR) using Benjamini–Hochberg correction (22). Event pairs that had a significant interaction term at FDR-corrected *P* < 0.05 were selected for sex-stratified analyses. All Cox regression models were adjusted for those known CHD-risk factors that were available in FinnGen: sex, birth year, smoking status, and body-mass-index (BMI). BMI was defined as weight (kg)/height^2^ (m^2^). Height and weight for BMI calculation were measured by nurses and smoking status was determined by self-report. We excluded from the analyses individuals that had outcome before baseline or had outcome event before the predictor event. We ignored the time for predictor events before the start of the study (if the predictor preceded the study start).

From sex-stratified Cox regression models a ratio of hazard ratios (rHR = HR women/HR men) was calculated for each predictor-CABG pairs and CABG-outcome pairs. For the main results reported in Tables 1, 2, we applied a clinical significance limit of rHR > 1.5 or rHR < 0.66 with *n* > 200 and associations outside these thresholds limits were only reported in the Supplementary Table 1. From the remaining traits, we further excluded similar and overlapping traits using most significant interaction *p*-value as the rule-in criteria.

**TABLE 1:** Predictors with sex related risk prior to coronary artery bypass grafting (CABG).

Predictor	Men (*n* = 4988)			Women (*n* = 962)			HR-ratio	Interaction *q*-value
		
	*n*	HR	*q*-value	*n*	HR	*q*-value		
Type 1 diabetes	145	3.2	7.0E−09	63	12.8	6.1E−17	4.0	5.1E−07
Thoracic aortic aneurysm	179	2.7	5.0E−07	23	15.6	3.9E−07	5.7	0.002
Other heart diseases	2186	4.8	3.3E−150	539	7.1	2.1E−83	1.5	0.003
Other chronic obstructive pulmonary disease	177	0.9	0.660	29	1.4	0.308	1.5	0.005
Diseases of the ear and mastoid process	442	0.8	0.006	138	1.4	0.023	1.8	0.027
Disorders of choroid and retina	259	1.2	0.102	101	2.4	1.1E−05	1.9	0.049

False discovery rate adjusted interaction *q*-value < 0.05 was considered statistically significant. 1.5-fold increase or decrease in HR and over 200 incidents in each predictor category was considered clinically significant. CABG, coronary artery bypass crafting; n = number of CABG patients with the predictor, HR ratio = women HR/men HR.

**TABLE 2:** Association of CABG and incident outcomes by sex.

Outcome	Men (*n* = 4988)			Women (*n* = 962)			HR-ratio	Interaction *q*-value
		
	*n*	HR	*q*-value	*n*	HR	*q*-value		
Hypertensive diseases (excluding secondary)	918	1.0	0.87	176	1.6	0.15	1.5	2.8E−05
Death due to cardiac causes	1471	2.5	3.1E−15	252	6.3	9.4E−08	2.5	8.2E−04
Alzheimer’s disease	347	1.6	0.006	82	4.2	7.3E−06	2.7	0.006
Other retinal disorders	381	1.3	0.022	112	4.1	3.7E−07	3.0	0.010
Thoracic aortic aneurysm	358	2.1	2.7E−09	34	6.1	3.8E−09	2.9	0.011
Metabolic disorders	1207	3.0	2.8E−16	274	7.5	9.6E−12	2.6	0.020
Gout	296	2.5	9.6E−11	35	5.8	1.3E−05	2.3	0.024
Gonarthrosis, primary, with knee surgery	182	1.1	0.47	40	0.5	0.04	0.5	0.027
Chronic kidney disease	405	2.7	3.3E−13	68	5.5	2.3E−06	2.0	0.032
Malaise and fatigue	424	2.3	9.7E−10	130	9.4	3.8E−17	4.0	0.039

False discovery rate adjusted interaction *p*-value < 0.05 was considered statistically significant. 1.5-fold increase or decrease in HR and over 200 incidents in each outcome category was considered clinically significant. CABG, coronary artery bypass crafting; n = number of CABG patients with the outcome, HR-ratio = women HR/men HR.

**FIGURE 1:**
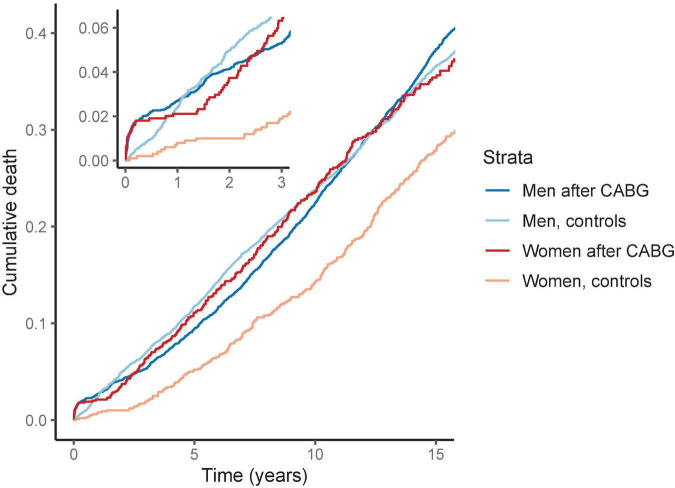
Cumulative death by sex after coronary artery bypass grafting (CABG). The survival curves are from Kaplan-Meier estimator. We matched the models for age, BMI and smoking status. The risk for death is elevated in women after CABG compared to matched women. In contrast, after 1 year men with CABG had similar risk of death than the matched men in the control group.

**FIGURE 2:**
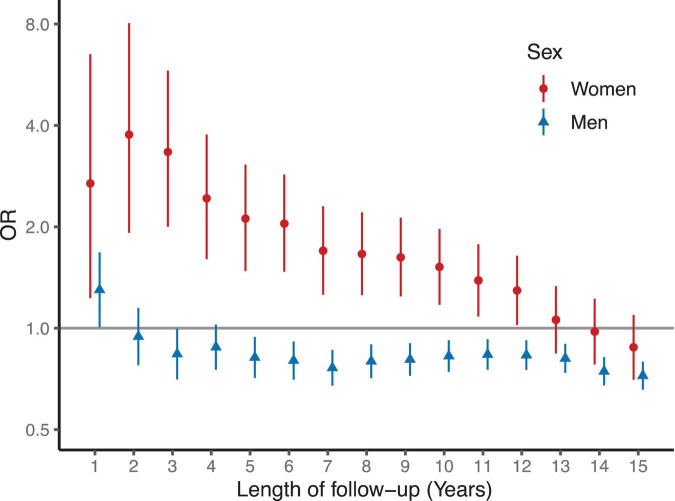
Odds ratios for death by sex after coronary artery bypass grafting (CABG). Logistic regression models are matched and adjusted for age, BMI and smoking status. Women had increased OR for any death for 12 years after CABG compared to matched and risk adjusted women. In contrast, men had similar or moderately reduced risk for any death than the men in the matched and risk adjusted control group.

To further investigate the impact of sex on all-cause of mortality, we used a case-control approach for men and women who underwent CABG. Each case (individuals who underwent CABG) was then matched with a control (individuals who did not undergo CABG) 1:1 for sex, birth year, BMI and smoking status using nearest neighbor matching with propensity score as distance. This resulted 999 cases and 999 controls for females and 5,222 cases and 5,222 controls for males. For cases, age of the first CABG was used as index age, while for controls index age was either age at year 1998 or 0, if birth year was after year 1998. An individual was included only if the end of follow up was after the index age. Using this dataset, we created Kaplan Meier curves (Figure 1) and logistic regression models (Figure 2) for cases and controls. Logistic regression models were performed separately for both sexes and for different follow up lengths.

Python 3.7.3. (lifelines library, Python Software Foundation, Beaverton, OR) and R 4.0.4 (The R Foundation, Vienna, Austria) was used for the statistical analyses.

## Results

### Patient characteristics

We studied 176,680 individuals (82,794 men and 91,568 women) from FinnGen database Data Freeze 7. The mean birth year of the sample was 1957.6 ± 18.4, 52.5% were women, 25.6% were active smokers, and the mean BMI was 27.4 ± 5.3 kg/m^2^. Mean BMI was 27.3 ± 5.8 kg/m^2^ in women, and 27.5 ± 4.6 kg/m in men, respectively. In total, 27,196 (32.8%) of men and 17,454 (19.1%) of women were past or current smokers. A total of 5,950 (4,988 men and 962 women) individuals underwent CABG surgery during follow-up period. The mean age at the time of the procedure was 64.7 ± 9.4 years and median follow-up time for CABG was 9.3 ± 6.1 years. From all potential 8,364 event pairs, we performed 1,192 analyses where trait preceded CABG procedure and 1,423 analyses where CABG procedure preceded trait (Supplementary Table 1). The ICD-codes for the observed predictor- and outcome traits are illustrated in more detail in Supplementary Table 2.

### Sex-differences in coronary artery bypass grafting-related morbidity

We observed five traits that were more strongly associated with future CABG in women than in men (Table 1). These traits were type 1 diabetes, thoracic aortic aneurysms, other heart diseases, other chronic obstructive pulmonary disease, diseases of the ear and mastoid process and disorders of choroid and retina.

We observed several sex differences in the associations between CABG and incident traits (Table 2). In general, the associations between CABG and incident traits (hypertension, cardiac death, Alzheimer’s, retinal disorders, thoracic aortic aneurysm, gout, chronic kidney disease, and fatigue) were greater in women than in men. CABG was only related to lower risk of knee arthrosis in women than in men.

### Sex differences in risk of death

The risk of cardiac death after CABG was 2.5- and 6.3-fold in men and in women as compared with controls of the same sex, respectively (Table 2). In the Kaplan–Meier survival analysis, the risk of short- and long-term death for women after CABG was higher than in the control group of matched women without CABG. In men, the Kaplan–Meier analysis demonstrated an increased cumulative risk for death during the first year after CABG but this difference in mortality was similar to the matched male control group during the rest of the follow up (Figure 1).

In the logistic regression analysis, an increased odds for all-cause death were observed in women with CABG compared to matched women during follow-up (Figure 2). The odds ratios for death were highest during the first 3 years after surgery and remained higher than in the control group for 12 years (Figure 1). In contrast, men after CABG had only modestly increased odds of death during the first year of follow-up, after which the odds of death were lower than in the control group of matched men.

## Discussion

In a systematic analysis of 5,950 CABG patients and over 1,100 different traits from the FinnGen study, we evaluated the sex-dependent differences in mortality and morbidity before and after CABG. In women, CABG was more strongly related to greater increases in risk of diseases such as hypertension, Alzheimer’s, aortic aneurysms, gout, and chronic kidney disease compared to risk increases observed in men. We observed that the risk for cardiac death was significantly higher in women after CABG compared to women who did not undergo CABG. The risk of cardiac death for men who underwent CABG was also higher than in the matched control population without CABG. However, the combined acute and long-term risk of death was 2.5-fold greater in women than in men, when compared to non-operated counterparts of the same sex.

Female sex has previously been associated with increased cardiovascular mortality and morbidity after CABG (4, 5, 23). Recent meta-analysis on sex differences in CABG outcomes from 2021 reported female sex to associate with higher risk of operative (odds ratio 1.77) and late mortality (incidence rate ratio, IRR 1.16) (5). Also, same study reported higher risk for major adverse cardiac event (IRR 1.40), myocardial infarction (IRR 1.28) and stroke (IRR 1.31) for women after CABG compared to men (5). Indeed, several previous studies have assessed the post-operative risk in women in relation to men. In our study, we assessed the association of CABG with mortality and morbidity separately for both sexes, with age-, BMI- and smoking status -matched controls. We observed that after CABG, men have a moderately elevated risk of death as compared to other men, whereas in women this risk is markedly higher (Figures 1, 2).

Sex differences in mortality after CABG are thought to be explained by a greater comorbidity burden in women. Both type 1 and 2 diabetes are associated with excess CVD risk in women as compared to men (10). In this study, we observed a statistically significant sex interaction with type 1 diabetes for future CABG (HR for men 3.2; HR for women 12.8; Table 1). Disorders of choroid and retina, which include diabetic retinopathy, were also more strongly associated with future CABG in women than in men. Furthermore, hypertension was observed to have sex-dependent interaction after CABG with 1.5-fold risk in women compared to men. The other traditional CVD risk factors, apart from hypertension, carried a similar risk for future CABG in both sexes. These findings therefore highlight the need for adequate control of diabetes and hypertension particularly in women to prevent future CABG.

Thoracic aortic aneurysms were observed to associate strongly with CABG in women both before and after CABG (Table 2). Male sex, old age, aortic atherosclerosis, hypercholesterolemia, genetic predisposition, smoking, and hypertension are risk factors that are known to associate with aortic aneurysm formation (24). However, CABG-operated women had a higher aortic aneurysm risk before and after CABG than in women without CABG. In men, however, these risks were much more modest. These findings suggest that aneurysm risk factors seem to potentiate more strongly in CABG-operated women than in men.

The similar results observed for COPD further emphasize the importance of smoking as a strong CABG risk factor in women (10). In this study, COPD was more strongly associated with future CABG in women than in men. It its known that COPD is a CVD risk factor due to common risk factors, such as smoking. Women with COPD have less cardiovascular comorbidities than men but conversely a more rapid COPD progression (25). Smoking is also a stronger risk factor for MI in women than in men (26). Thus, the observed sex differences in the COPD-CABG associations may be a result of their shared risk factors, thus highlighting the importance of smoking cessation in women undergoing revascularization (27).

Disorders of the choroid and retina were more strongly related to risk of CABG in women than in men. This relation was similar when CABG was the predictor variable. This miscellaneous category included phenotypes, such as macular degeneration, retinal breaks, diabetic retinopathy, maculopathy, and preretinal fibrosis, which all share risk factors with CVD. Smoking and age are risk factors for macular degeneration in both women and men, but obesity, hypertension, and low physical activity are associated with increased risk of macular degeneration only in women (28). Diabetic retinopathy is the most common microvascular complication of diabetes and is more common in men (29). However, as diabetes is a more potent risk factor for death and cardiovascular disease in women (30), the observed association between retinal disorders and future CABG is likely to represent the effects of diabetes and smoking.

We also observed that diseases of ear and mastoid process were associated with future CABG particularly in women. This phenotype consists of several miscellaneous diseases such as sensorineural hearing loss, benign paroxysmal vertigo, and infections of the outer and middle ear. Hearing loss is known to associate with atherosclerosis and cardiovascular disease with a possible underlying mechanism of microvascular impairment (31, 32). This association is stronger in women than in men (31). Therefore, the increased risk for CABG in women with sensorineural hearing loss may be considered a surrogate of CVD, particularly with concomitant retinopathy.

CABG was also associated with several other incident traits after the operation. For instance, CABG was a stronger risk factor for Alzheimer’s disease in women than in men. This is could be explained by the common risk factor profiles associated with CVD and Alzheimer’s, including hypertension, diabetes and obesity (27). Another possibility could be that women are more susceptible to cognitive impairment caused by the operation and the anesthesia themselves.

Coronary artery bypass grafting was found to associate with incident gout more strongly in women than in men (Table 2). Hyperuricemia, an increase of uric acid in circulation, is required for gout, but hyperuricemia also independently associates with coronary artery disease and other CVD, especially in elderly women (33). However, results have been conflicting as other studies consider hyperuricemia as a risk factor while other studies have not found any association with these two conditions (33). Our finding could be explained by sex differences in medications, such as diuretics, and comorbidities.

Coronary artery bypass grafting in women was associated with incident CKD and the risk was 2.0-fold compared to men. Acute kidney injury is common immediately after CABG and also a risk factor for progression to later CKD and increases morbidity and CVD mortality (34). This sex-dependent observation may be one factor in explaining the worse outcomes of women after CABG. The reasons underlying this finding remain unclear and warrant further research.

History of CABG in women was more strongly associated with incident metabolic disorders, comprised of hypercholesterolemia, hyponatremia or hypokalemia, in women than in men. As expected, CABG was associated with increased risk of hypercholesterolemia after CABG. However, this risk was greater in women than in men. This association most likely reflects that women are more susceptible of post-operative electrolyte disorders and less aggressive lipid-lowering therapy.

Further, CABG was related to a reduced risk of knee replacement surgery in women compared with men. The research on the relation of osteoarthrosis and CVD has provided conflicting results. Some publications have suggested that individuals with osteoarthrosis, and particularly women, have an increased CVD risk (35–37), whereas others studies have not found an association between CVD and knee osteoarthritis (38). The possible mechanisms behind the link between osteoarthritis and CVD could be related to shared causal factors, such as vascular inflammation and microvascular changes (39). However, the causes underlying the observed sex differences warrant further study, although is possible that women with a history of CABG are less often referred to surgical interventions than men.

Finally, the phenotype other heart diseases was observed to associate more robustly with future CABG in women than in men. This phenotype includes various disease entities such as heart and valve infections, cardiomyopathies, conduction disorders, arrythmias, and various types of heart failure. This heterogeneity of the diagnoses renders further interpretation of the observed association challenging. Similarly, the phenotype malaise and fatigue, with an HR of 9.4 in women after CABG, is a very unspecific diagnosis, but this finding may represent poorer control of symptoms in women.

A limitation and strength of this untargeted epidemiological study is the large number of different traits available for statistical analysis. Despite adjustments for age, BMI and smoking status, the unavailability of exact measurements of lipid-, glucose- and blood pressure levels prevents a more detailed assessment of individual risk profiles. Also, detailed information on ejection fraction, number of coronary bypasses, and the acuity of surgery were not available in this register-based study. Furthermore, as in other studies, the number of women who underwent CABG was low compared to that of men. To address these potential biases, we adjusted for multiple testing and used clinically significant thresholds of HR > 1.5 or HR < 0.66 for the main results. Moreover, as statistical associations do not indicate causality, more research is needed in order to discover the mechanisms of the observed connections to improve the outcomes in women with CHD.

## Conclusion

Women are at higher risk for diseases after CABG, which further increase the risk of death. After CABG, the relative risk of long-term death in women is significantly higher than in men. Consideration should be given to whether women receive adequate treatment post-CABG, as CABG does not improve life expectancy in women as much as in men.

## Data availability statement

The FinnGen data may be accessed through Finnish Biobank’s Finngenious portal at www.fingenious.fi and at www.FinnGen.fi/en.

## Ethics statement

Patients and control subjects in FinnGen provided informed consent for biobank research, based on the Finnish Biobank Act. Alternatively, separate research cohorts, collected prior the Finnish Biobank Act came into effect (in September 2013) and start of FinnGen (August 2017), were collected based on study-specific consents and later transferred to the Finnish biobanks after approval by Fimea (Finnish Medicines Agency), the National Supervisory Authority for Welfare and Health. Recruitment protocols followed the biobank protocols approved by Fimea. The Coordinating Ethics Committee of the Hospital District of Helsinki and Uusimaa (HUS) statement number for the FinnGen study is Nr HUS/990/2017. The FinnGen study was approved by Finnish Institute for Health and Welfare (permit numbers: THL/2031/6.02.00/2017, THL/1101/5.05.00/2017, THL/341/ 6.02.00/2018, THL/2222/6.02.00/2018, THL/283/6.02.00/2019, THL/1721/5.05.00/2019, and THL/1524/5.05.00/2020), Digital and population data service agency (permit numbers: VRK43431/2017-3, VRK/6909/2018-3, and VRK/4415/2019-3), The Social Insurance Institution (permit numbers: KELA 58/522/2017, KELA 131/522/2018, KELA 70/522/2019, KELA 98/522/2019, KELA 134/522/2019, KELA 138/522/2019, KELA 2/522/2020, and KELA 16/522/2020), Findata (permit numbers: THL/2364/14.02/2020, THL4432/14.06/2020, THL66 19/14.06.00/2020, THL/1284/1406.00/2021, THL/4055/14.06. 00/2020, THL/3433/14.06.00/2020, THL/5189/14.06/2020, TH L/5894/14.06.00/2020, THL/209/14.06.00/2021, THL/688/14.06. 00/2021, THL/1965/14.06.00/2021, and THL/5546/14.02.00/2020), Statistics Finland [permit numbers: TK-53-1041-17 and TK/143/07.03.00/2020 (earlier TK-53-90-20)]. The Biobank Access Decisions for FinnGen samples and data utilized in FinnGen Data Freeze 7 include: THL Biobank BB2017_55, BB2017_111, BB2018_19, BB_2018_34, BB_2018_67, BB2018_71, BB2019_7, BB2019_8, BB2019_26, BB2020_1, Finnish Red Cross Blood Service Biobank 7.12.2017, Helsinki Biobank HUS/359/2017, Auria Biobank AB17-5154 and amendment #1 (August 17 2020), Biobank Borealis of Northern Finland_2017_1013, Biobank of Eastern Finland 1186/2018 and amendment 22 §/2020, Finnish Clinical Biobank Tampere MH0004 and amendments (21.02.2020 & 06.10.2020), Central Finland Biobank 1-2017, and Terveystalo Biobank STB 2018001. The patients/participants provided their written informed consent to participate in this study.

## Author contributions

JN wrote the original draft and revisions. AK and JP analyzed and interpreted the patient data. JA and TN planned, reviewed, and edited the manuscript. All authors contributed to the article and approved the submitted version.
